# Survival-Span Method: How to Qualitatively Estimate Lifespan to Improve the Study of Aging, and not Disease, in Aging Studies

**DOI:** 10.3389/fragi.2021.724794

**Published:** 2021-12-14

**Authors:** Julia Adelöf, Jaime M. Ross, Madeleine Zetterberg, Malin Hernebring

**Affiliations:** ^1^ Department of Clinical Neuroscience, Institute of Neuroscience and Physiology, Sahlgrenska Academy at the University of Gothenburg, Gothenburg, Sweden; ^2^ Department of Biomedical and Pharmaceutical Sciences, College of Pharmacy, George & Anne Ryan Institute for Neuroscience, University of Rhode Island, Kingston, RI, United States; ^3^ Department of Neuroscience, Biomedicum, Karolinska Institutet, Stockholm, Sweden; ^4^ Department of Ophthalmology, Region Västra Götaland, Sahlgrenska University Hospital, Mölndal, Sweden

**Keywords:** aging, lifespan, survival analysis, animal research, 3R

## Abstract

Lifespan analyses are important for advancing our understanding of the aging process. There are two major issues in performing lifespan studies: 1) late-stage animal lifespan analysis may include animals with non-terminal, yet advanced illnesses, which can pronounce indirect processes of aging rather than the aging process *per se* and 2) they often involves challenging welfare considerations. Herein, we present an option to the traditional way of performing lifespan studies by using a novel method that generates high-quality data and allows for the inclusion of excluded animals, even animals removed at early signs of disease. This Survival-span method is designed to be feasibly done with simple means by any researcher and strives to improve the quality of aging studies and increase animal welfare.

## Introduction

Aging is a complex process that affects virtually all organisms and tissues, and is a major risk factor for developing diseased states, such as forms of cancer, cardiovascular diseases, and neurodegenerative diseases. Thus, a better understanding of the mechanisms of aging is of major importance for most, if not all, medical fields (Harman, 1991; Niccoli and Partridge, 2012). Aging studies in animal models are complex, expensive, and require optimal study design and execution in order to obtain useful data to draw reliable conclusions. Determining lifespan is an important analysis used in conducting aging studies and aids in identifying the molecular mechanisms that control the pace of aging, including drug intervention, the effect of a gene, as well as lifestyle factors such as diet (caloric restriction, high-fat diet etc.) and exercise. However, experiments that monitor and test animals until advanced age, such as lifespan and behavioral studies, are challenging to execute due to the fact that the aging process strongly correlates with disease onset and progression. Thus, it is often the case that studies of aging also include diseased animals, and analyses on these animals consequently investigate both aging and disease, which in turn could introduce subject variation and conceal mechanistic changes brought about by the aging process *per se*. This is particularly important in homogenous cohorts, for example inbred mice, that are more prone to develop specific diseases due to homozygosity rather than heterogeneous populations such as humans. Thus, by excluding animals that show overt signs of decreased health, variance within the remaining cohort is less likely due to an effect of illness or disease*.* Although necropsies can provide clarity as to the health state of an animal, insights from this postmortem approach cannot be applied *ex post facto.* This quandary has led the quest for better discernment between aging versus disease. From a study design perspective, however, the removal of diseased animals from a lifespan analysis can be problematic because it: 1) selects for some aged animals, and not all aged animals, 2) decreases the power of the study, and 3) might provide misleading data on actual aging.

In a previously published behavioral aging study, we devised a novel method that can be used for lifespan analysis, herein coined “Survival-span method”. This method allows for the inclusion of euthanized animals together with animals that died from “natural causes” (Adelöf et al., 2019). The Survival-span method generates high-quality data on survival using the entire data set and without reducing the number of animals, skewing results, or compromising animal welfare. Our approach involves creating a range between minimum and maximum survival curves by categorizing removed animals differently in the Kaplan-Meier survival analysis. The minimum survival curve is generated by labelling the removed animals similarly to animals that died from “natural causes”, which creates an underestimation of natural lifespan since those animals would have lived longer, although diseased, if not removed. The maximum survival curve is then generated when the removed animals are instead “censored” (i.e., a statistically unknown fate when removed from the study), which generates an overestimation of lifespan since it considers these animals as merely removed from the study at a given time. The actual lifespan lies in the span between these two survival curves.

The Survival-span method was first performed using hybrid mice in our previous study, though this method is applicable to all animal lifespan analyses. The interval of median lifespan in our C57BL/6N×BALB/c F2 male hybrids was very similar (789 ± 130 compared to 742–826 days) to the four-way cross UM-HET3 male mice (offspring of BALB/cByJ×C57BL/6J F1 females and C3H/HeJ× DBA/2J F1 males), and was slightly lower in females (801 ± 88 compared to 832–891 days; Strong et al., 2013; Adelöf et al., 2019). In comparison to inbred mice, the Survival-span of the C57BL/6N×BALB/c F2 hybrids surpassed that of short-lived BALB/c, but did not attain the longevity of the long-lived C57BL/6J mice, using data from the Aging Phenome Project (711/901 days for males and 771/866 days for females; Yuan et al., 2009; Adelöf et al., 2019). The Survival-span method was also recently applied in understanding the effect of overexpressing the proteasome activator PA28αβ (Adelöf et al., 2021).

Lifespan analyses are important in determining the effect of a gene as well as efficacy of drug interventions and other therapeutic approaches to elucidate the underlying molecular mechanisms of aging. Herein, we present a step-by-step description of how to generate lifespan analyses applying the Survival-span method. Using the Survival-span method allows for the inclusion of all animals in the study while removing those with advanced diseased states and provides distinction between animals that present healthy from those with decreased health. Additionally, the Survival-span method decreases the introduction of sample variance due to disease-associated alterations that could confound behavioral/biochemical analyses of the aging process and creates results that are comparable to other aging studies. Lastly, this method improves animal welfare and facilitates institutional compliance while performing lifespan analyses, even under more rigorous animal guidelines.

## Material and Equipment

The requirements to perform this method are a spreadsheet program (e.g., Microsoft Excel) and a statistical program (e.g., SPSS, SAS, Prism) in which Kaplan-Meier survival analysis can be done. The Kaplan-Meier nonparametric test revolutionized survival analyses when presented in 1958 because it allowed, for the first time, the inclusion of incomplete observations in survival statistics. To correctly run the Kaplan-Meier analysis, specific criteria (statistically coined “assumptions”) regarding the dataset need to be acquired as indicated below (Kaplan and Meier, 1958).

1) The time until observation for each subject must be clearly defined and precisely measured. Having a clear starting point is optimal to reduce the risk of left-censoring, which can skew results. For example, if survival time of a specific subject group is studied, left-censoring can be a result of inclusion criteria dependent on vague diagnosis, making the actual starting point difficult to obtain. In lifespan studies, left-censoring is not an issue if the animal’s exact birth date is used.
2) Every observation must fit into one of the two different states: “event” or “censored”. In lifespan studies, the observation is animal fate and can be either “event” (death) or “censored” (removed from study).
3) The two states should be independent of each other and subjects should not be categorized as “censored” if the risk of an “event” occurring is increased. The Kaplan-Meier analysis assumes that censored data behaves as uncensored data beyond the time of censoring and if the prognosis of censored subjects relates to the event, this introduces a bias. However, in aging studies this assumption is difficult to adhere to since diseased subjects have an increased risk of dying and should not be either censored or removed from the study. The Survival-span method addresses this bias by including both possible fates and not selecting one fate above the other. Using both possible fates results in two lifespan calculations, one being an underestimation and the other an overestimation of an animal’s “true” lifespan.

To evaluate if the survival curves of experimental groups are significantly different, there are a variety of statistical tests that can be applied. The most commonly used is the log-rank (Mantel-Cox) test, but depending on the dataset other tests may be more appropriate. The log-rank test is nonparametric and calculates the chi-square (χ^2^) for each group for each event time and summarizes the results. Similar to the Kaplan-Meier test assumptions, the log-rank test requires that the comparison groups include similar degrees of censoring and that survival probability is the same regardless of when subjects were included in the study. The log-rank test should not be used on datasets with overlapping survival curves (Mantel, 1966; Cox, 1972; Peto and Peto, 1972; Bland and Altman, 2004). In contrast to the log-rank test, which weighs all calculations equally, the Wilcoxon (also called Breslow, Gehan) test weighs early events heavier than late ones and may be a preferred test to use when the initial phase is of particular importance and censoring is scarce (Breslow 1974).

## Methods

To perform the Survival-span method, follow these instructions. Steps 1–3 are done in a spreadsheet (e.g., Excel), and steps 4–5 in a statistical program (e.g., SPSS, SAS, Graphpad Prism).

1) Procedure: For each subject, log the date of birth and the number of days the birth date differs from the start of the study, as shown in Table 1. Number the days of the study, as shown in Table 2.

**TABLE 1:** Date of birth log.

Animal number	Date of birth	Days from birth to start of the study
350	April 28, 2020	−3
357	May 1, 2020	0
366	May 4, 2020	+3
…		

**TABLE 2:** Day of study log.

Date	Study day
May 30, 2020	30
May 31, 2020	31
June 1, 2020	32
June 2, 2020	33
…	

Rationale: To adhere to the assumption of exactness of time until an event is recorded, it is important to log the date of birth and days of lifespan. At the time of starting a lifespan study, animals typically would not be born on the same day. Thus, one should register each animal’s date of birth and the number of days differing for all animals to obtain a precise time until fate. In addition, a reference calendar for days of the lifespan study should be created. This enables knowing the exact study day for each animal.

2) Procedure: Monitor animals on a regular basis and remove animals with significantly decreased health.

Rationale: Follow the animal research permit of the study per institutional guidelines. If using mice, animals should be checked at least twice per week when less than 24 months of age, and daily when older than 24 months of age. Symptoms indicating significant decreased health in mice may include, for example: hunched shoulders, shabby fur, rapid decrease in activity, decreased eating/drinking, tumors.

3) Procedure: Log the fate of each animal in the lifespan study. Code animals that died from “natural causes” with “1” for both minimum and maximum survival boxes. Euthanized animals are coded with “1” in the minimum survival log and with “0” in the maximum survival log, as shown in Table 3.

**TABLE 3:** Generation of minimum and maximum survival codes.

Animal number	Birth date difference (birth date –study start date)	Study day of fate	Lifespan (study days –birth date difference)	Fate	Minimum survival code *1= all deaths*	Maximum survival code
						*1= natural*
						*0= euthanized*
366	+3	903	900	Natural	1	1
357	0	957	957	Euthanized	1	0
350	−3	957	960	Natural	1	1
…						

Rationale: To prepare data for statistical analysis, the fates need to be converted into “1”and “0” so that statistical programs can run the Kaplan-Meier analysis. In the Kaplan-Meier analysis “1” signifies an event and “0” signifies censored subject. It is the coding of the euthanized subjects that differs in the two lifespan curves.

4) Procedure: Transfer Table 3 data (use columns: Lifespan for each animal with both Minimum and Maximum Survival Codes) to an appropriate statistical program where Kaplan-Meier analysis can be performed. Set Lifespan as time (X-variable) and Minimum and Maximum survival as two separate columns or groups (Y-variables), as shown in Table 4.

**TABLE 4:** Data transfered into a statistical program.

Days of lifespan (X)	Minimum survival (Y_1_)	Maximum survival (Y_2_)
900	1	1
957	1	0
960	1	1
…		

Rationale: Enables a statistical program to run the Kaplan-Meier analysis.

5) Procedure: Run the statistical analyses and obtain results.

**FIGURE 1:**
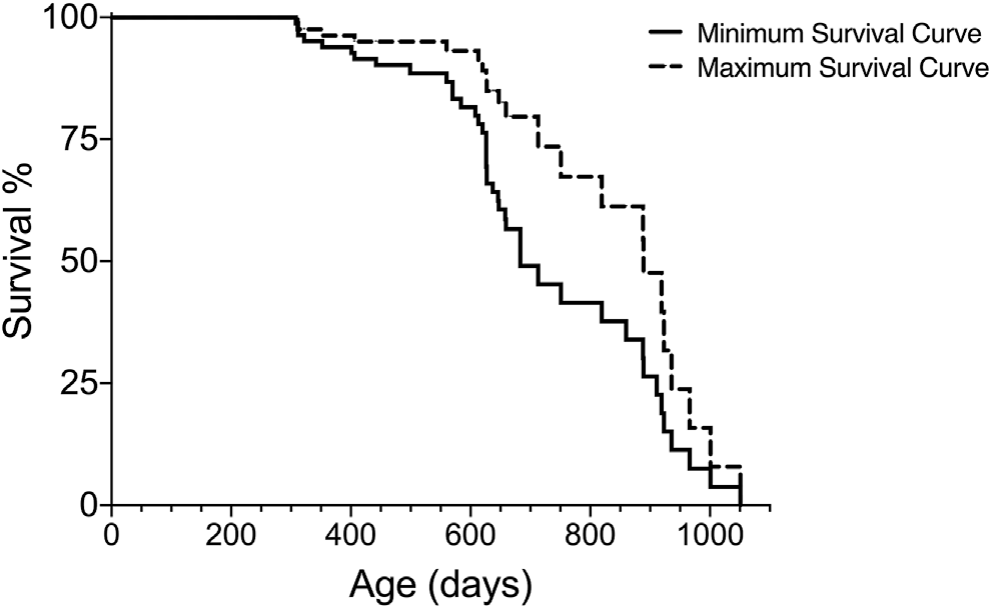
Graphical readout of minimum and maximum survival curves generated with Kaplan-Meier analysis for one group of subjects.

Rationale: The Kaplan-Meier Survival analysis will generate data of survival (e.g., median, standard deviation of the median, % survival, # of subjects for each fate) and graphs for visualization of lifespan (see Figure 1). Some statistical programs also report estimates of the mean survival; however, this should be analyzed with caution since it does not take the censored subjects into account and may be misleading.

**FIGURE 2:**
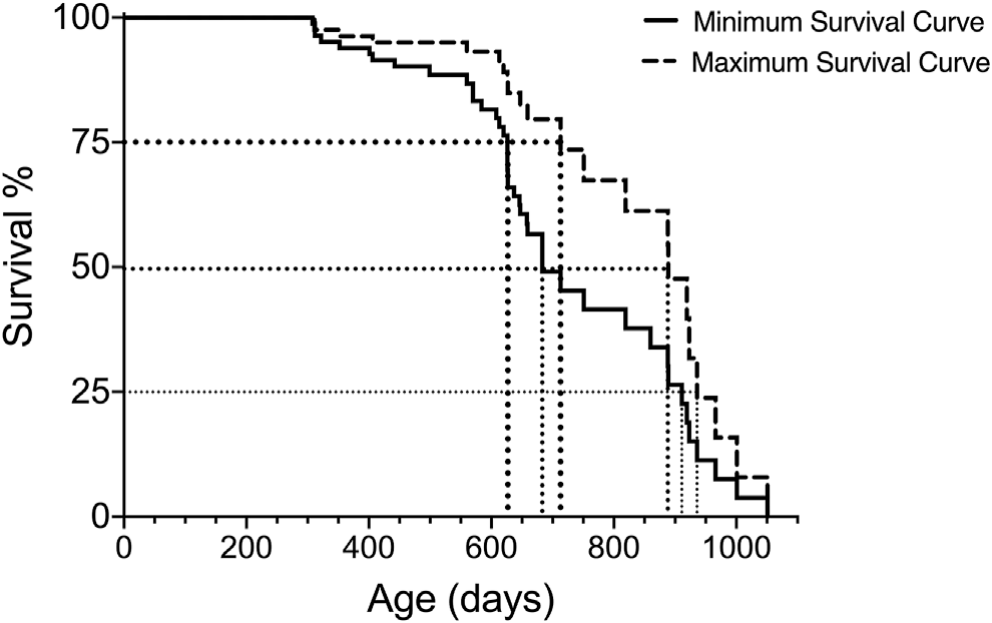
Extrapolation of 75, 50 and 25% survival for both minimum and maximum survival curves generated with Kaplan-Meier analysis for one group of subjects.

In addition to the median (50%) survival, it can be helpful to estimate additional survival times (e.g., 25%, 75%) in order to compare the lifespan data with other aging studies. These additional survival percentages can be extrapolated from the Kaplan-Meier analysis and visualized as shown in Figure 2 and presented in Table 5.

**TABLE 5:** Extrapolated 75, 50 and 25% survival for both minimum and maximum survival curves in Figure 2.

	75% survival (Days)	50% (median) survival (Days)	25% survival (Days)
Minimum Survival Curve	628	683	911
Maximum Survival Curve	714	889	936
Actual Lifespan	628–714	683–889	911–936

## Results

**FIGURE 3:**
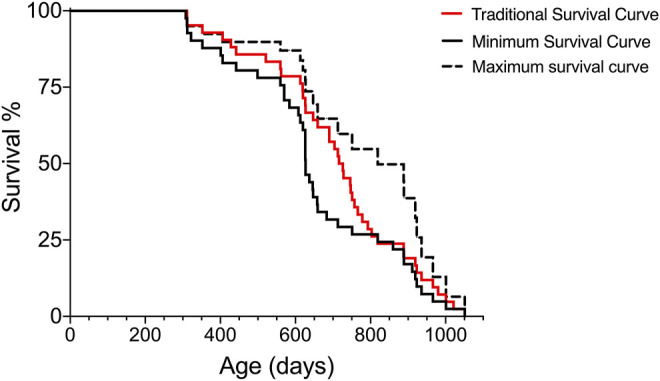
Hypothetical comparison of a traditional survival curve with minimum and maximum survival curves generated by the Survival-span method. Subjects in the hypothetical “traditional” survival analysis have been generated by adding 120 days to the euthanized subjects in the data set of Figure 1. The 120 days addition is an estimated average of potential extended survival.

In traditionally conducted lifespan studies, the strategy to generate a lifespan with the closest proximity to the exact lifespan is to, ideally, not interfere with the fate of subjects, but this may introduce variations due to disease and may not be compatible with ethical considerations of animal welfare. During aging, variation in cohorts of animals can increase; however, the removal of animals with evident signs of decreased health assists in decreasing some of the variation introduced by the reduced health state. Using the Survival-span method, all animals are included in the lifespan analyses regardless of when they might be removed due to decreased health. In Figure 3, we compare the lifespan curves using the Survival-span method with a hypothetical traditional survival curve to mimic a typical lifespan study. The hypothetical traditional survival curve was generated using the same data set shown in Figure 1, with 120 days added to all removed subjects as an estimate of how long they could have potentially lived if they would not have been euthanized (120 days was estimated as a rough average). The hypothetical traditional lifespan curve lies, as predicted, within the span of the maximum and minimum lifespan curves generated by the Survival-span method (Figure 3).

**FIGURE 4:**
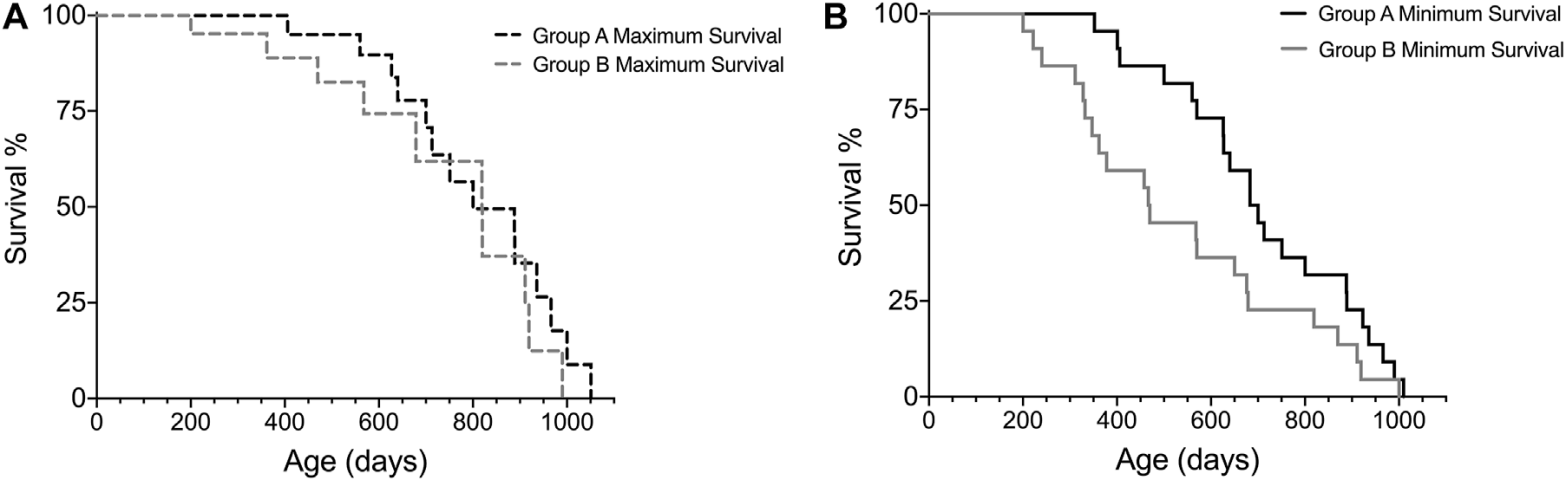
Hypothetical example on how the Survival-span method adds valuable information on survival. Subjects in group B tend to have a higher prevalence of early ill health causing early euthanization (∼200–300 days of age), which results in no difference in **(A)** maximum survival, but a significant difference in **(B)** minimum survival, when comparing group A and B (*p* = 0.0438, Log-rank test) (n_groupA_ = 22, n_groupB_ = 22, censored_max-curveA_ = 9, censored_max-curveB_ = 9).

For comparison of survival intervals between different groups, we recommend performing a log-rank test (if Kaplan-Meier assumptions are met) for all minimum survival curves amongst cohorts and then repeating these analyses for all maximum survival curves. Analyzing the minimum and maximum survival curves separately allows for adhering to the assumption of similarity of censoring. In addition, comparing the minimum and maximum survival curves can result in valuable lifespan observations, such as if there were significant differences in minimum survival but not maximum survival, as illustrated in Figure 4 and Table 6. This is a hypothetical example of a scenario where there is no difference between groups when considering only subjects that reach old age and die of “natural causes”, as shown by the maximal survival curves of group A and B (Figure 4A), but subjects in group B are more likely to have earlier onset of ill health, as indicated by the minimal survival curves (Figure 4B). If the animals in group B would not have been euthanized, they would most likely have died from “natural causes” earlier than subjects in group A. Thus, only looking at one survival curve when conducting an aging study could yield an analysis indicating that group B has a shorter lifespan than group A, which might be interpreted as a difference in the aging process. The same scenario, however, presented using our Survival-span method, highlights that disease states, and not necessarily aging *per se*, skew lifespan of group B, since in this example the subjects in group B that do not die early from disease are in fact just as long-lived as the subjects in group A.

**TABLE 6:** Comparison of experimental groups A and B survival curves by log rank test.

Log-rank values	Minimal survival curves	Maximal survival curves
χ^2^	4.064	2.870
df	1	1
*p*	0.0438	0.0903

The total number of subjects in this example is 22 for each group, in the maximal survival curves analysis, nine subjects/group have been censored.

In general, researchers need to have clear and consistent censorship criteria when conducting animal lifespan studies. These criteria become even more important for survival analyses when applying the Survival-span method. Causes for euthanization may differ depending on the animal species, disease model, therapeutic treatment, researcher, institution, or country, all which can affect outcomes of the Survival-span method, highlighting a possible limitation of the method. We thus recommend researchers to standardize the criteria for the removal of animals in their lifespan studies. Notably, even in animals that appear healthy, full necropsy can show otherwise, thus the existence of disease can be concealed in animals that are not removed. A limitation related to the removal of animals is reflected in disease presentation that could potentially introduce removal inconsistencies. For example, an animal with a visible tumor may be euthanized earlier than an animal with an internal tumor, with the latter possibly not euthanized until signs of secondary effects from the tumor. In this scenario, these two animals would most likely be removed from the study at different degrees of disease. Taken together, the limitations of the Survival-span method can be considered relatively minor, but important to take into consideration.

## Discussion

Lifespan studies are a crucial element to aging research and have been used to identify genes or pathways important in the regulation of lifespan, such as insulin/insulin-like growth factor 1 (IGF-1) signaling (Kenyon et al., 1993), mitochondrial DNA (mtDNA) mutation load (Trifunovic et al., 2004; Ross et al., 2013), as well to test drug intervention, such as rapamycin (Harrison et al., 2009) and metformin, and lifestyle factors, such as dietary restriction (reviewed in Fontana et al., 2010). The most commonly used statistical test to analyze lifespan is Kaplan-Meier survival analysis. Although statistical tests, like Kaplan-Meier, are essential for good experimental practice, it is sometimes difficult to fit biology into statistical analysis models. Due to the impossible compliance of the Kaplan-Meier assumption of bias, it is very challenging to generate an exact lifespan curve while adhering to animal health and welfare. In addition, the intertwining of aging with disease onset and progression further complicates the analysis of aging cohorts. Since statistics do not take into account fundamental insights of aging, it is important for researchers to try to modify statistical tests into models that serve the purpose of providing truthful scientific results as well as ethical considerations.

Here, we present a novel approach to the Kaplan-Meier survival analysis, the Survival-span method, which generates a lifespan analysis model to address these central issues of aging studies. Our method utilizes the assumption of independence for censoring of events (Kaplan-Meier assumption 3), and allows removed subjects to be designated as either “natural death” or “censored”, which creates an under- and overestimation of true lifespan. Importantly, and as demonstrated by examples presented herein and recently published (Adelöf et al., 2019), the Survival-span method still allows for qualitative comparisons with other aging studies, irrespective of where the research was conducted, and can also be used to assess differences between experimental groups (e.g., Figure 4; Table 6). In addition, allowing for the inclusion of all enrolled animals, even those removed for decreased health, adds valuable information about the cohort, which in turn improves translatability to humans.

Considering that aging and disease are tightly associated, it is not straightforward as how to, or if one should try to, distinguish between these two parameters in survival and aging analyses. Nonetheless, including animals with significant decreased health in an aging study yields variation that may reflect differences of the diseased states and not actually the aging process *per se,* which could affect conclusions of studies applicable to humans. In addition, how the removal of animals is statistically maneuvered impacts lifespan studies aiming to understand molecular mechanisms of aging. The Survival-span method is the first model to integrate an effect of decreased health into a survival analysis and this gives important biological information which may have been previously overlooked. For example, a recent aging study reported that overexpression of SIRT6, but not SIRT1, extended healthy lifespan; however, the mice that were euthanized were considered equal to mice that died from “natural causes” (Roichman et al., 2021). In this study, it is not stated how many or which mice were removed. Interestingly, autopsies at the time of “natural death” showed that the incidence of gastrointestinal adenoma was approximately 60% in SIRT1 overexpressing (SIRT1-OE) mice as compared to 30% in wild-type (WT) mice. The gastrointestinal adenoma induced in the SIRT1-OE mice is most likely independent of aging since the percentage of disease was not reflected in the WT mice. Given the high incidence, removal of SIRT1-OE mice may therefore be a result of disease at old age and not aging *per se*. Since the lifespan analysis in this study made no distinction between removed mice and mice that died from “natural causes”, any disease induced by SIRT1 overexpression is concealed but nonetheless shortens the survival curve of the SIRT1-OE cohort. Consequently, the outcome of SIRT1 overexpression, which the authors address as effects on lifespan, most likely also includes an effect of disease progression, independent of aging. Thus, it is mechanistically possible that a SIRT1 effect on healthy aging was undetected because it was counteracted by disease induction in this cohort. This example elucidates that lifespan analyses, as they are often conducted, may incorrectly assign results of lifespan interventions to aging, although in fact they can be independent of aging.

Using the Survival-span method may also provide valuable insights into sex differences in aging studies. In a recent study aiming to investigate the effect of restricting dietary branched-chain amino acids (BCAAs) on lifespan, the authors showed an increase on lifespan and reduction of frailty in male, but not female mice (Richardson et al., 2021). In our experience, a sudden drop in a survival curve (as shown in Figures 1A, 4A, 5D in Richardson et al., 2021) can signify incidence of disease, as demonstrated by our example shown in Figure 4; thus, employing the Survival-span method could have helped to differentiate between sex-specific BCAA-associated effects of disease incidence (minimum survival curve) and of aging (maximum survival curve) in Richardson et al., 2021. Hence, using the Survival-span method allows for visualization and measurements of findings that may be otherwise interpreted incorrectly and/or not discovered.

In addition to distinguishing decreasing health due to diseased states from actual aging, using the Survival-span method also promotes animal welfare. Beyond the field of aging, this method can be applied to any study across disciplines, such as the effects of various treatments on cancer. We hope that this method will be used as a standard for survival estimation in future lifespan studies as it provides a way to: 1) include all enrolled animals, 2) perform lifespan analysis while considering animal welfare, and 3) increase the quality of behavioral/biochemical analyses of the aging process.

## Data Availability

The original contributions presented in the study are included in the article/Supplementary Material, further inquiries can be directed to the corresponding authors.
